# Rapid genetic and ecological differentiation during the northern range expansion of the venomous yellow sac spider *Cheiracanthium punctorium* in Europe

**DOI:** 10.1111/eva.12392

**Published:** 2016-08-17

**Authors:** Henrik Krehenwinkel, Dennis Rödder, Magdalena Năpăruş‐Aljančič, Matjaž Kuntner

**Affiliations:** ^1^ Max Planck Institute for Evolutionary BiologyPlönGermany; ^2^ Environmental Science Policy and ManagementUniversity of California BerkeleyBerkeleyCaliforniaUSA; ^3^ Zoologisches Forschungsmuseum Alexander KoenigBonnGermany; ^4^ Transdisciplinary Research Centre Landscape – Territory – Information SystemsCeLTISUniversity of Bucharest Research InstituteICUBBucharestRomania; ^5^ Tular Cave LaboratoryKranjSlovenia; ^6^ Evolutionary Zoology LaboratoryBiological Institute ZRC SAZULjubljanaSlovenia

**Keywords:** adaptation, global change, isolation by environment, range expansion, venomous spider

## Abstract

Although poleward range expansions are commonly attributed to global change, a complex interaction of ecological and evolutionary factors might contribute to expansion success. Here, we study the expansion of the yellow sac spider *Cheiracanthium punctorium*, a medically important species in Central Europe. Using microsatellite markers and DNA sequences, morphological and climate niche analyses, we identify factors associated with the spider's expansion success. Our results indicate that the species' initial expansion has been triggered by environmental change and preadaptation in the source populations. However, despite extensive gene flow, expanding populations maintain genetic and morphological differentiation from native ones, which is correlated with climatic niche differences. Moreover, expanding spiders might have temporarily escaped an eggsac parasite that causes high mortality in the native range. Hence, our results paint a complex picture of diverse factors associated with expansion success. We speculate that expanding populations might be capable of adapting to novel ecological conditions in northern Europe. This could allow a substantial range expansion, much farther than by environmental change alone. Our distribution model predicts that the spider will soon massively spread over most of northern Europe, bringing along considerable health concerns.

## Introduction

Global ecosystems are currently exposed to unprecedented environmental change. Although global change is often associated with range contractions or even extinctions (Thomas et al. 2004), some species emerge as profiteers and expand their range into newly emerging habitat (Le Roux and McGeoch 2008). Such expansions are usually interpreted as responses to changing environmental conditions at the range edge (Hickling et al. 2006). Moreover, they could simply be the result of phenotypic plasticity allowing species to exploit changing environmental conditions (Gienapp et al. 2008; Merilä and Hendry 2014) or could even be affected by bacterial infections (Goodacre et al. 2009). However, the potential for a successful expansion is not necessary inherent to a species, but could also be acquired by evolutionary adaptation (Parmesan 2006; Hill et al. 2011). While environmental change could initially trigger range expansions, only the interplay with adaptation to, for example, novel climatic conditions at the range edge may lead to massive expansions as currently observed for many species (Clements and Ditommaso 2011; Franks and Hoffmann 2012). An expanding population might initially be released from selection pressure by competitors, parasites, and predators. This release has been shown in many invasive species and might speed up the population's regrowth and facilitate an adaptation to novel environmental conditions in a new habitat (Keane and Crawley 2002; Menéndez et al. 2008; Phillips et al. 2010). After the evolution of novel phenotypes, for example, increased physiological tolerance, a population can further expand its range, much farther than by environmental change alone (Clements and Ditommaso 2011; Franks and Hoffmann 2012). Indeed, several examples of rapid adaptive divergence during contemporary range expansions and biological invasions have been observed (Huey et al. 2000; Phillips et al. 2006; Colautti and Barrett 2013; Krehenwinkel et al. 2015). Even in the face of considerable ongoing gene flow between expanding and native populations, strong selection can mediate adaptations (Saint‐Laurent et al. 2003). Gene flow and secondary contact might even provide expanding populations with novel variation and facilitate adaptive differentiation (Rius and Darling 2014). In fact, genetic signatures of isolation of populations by environmental conditions appear to be quite frequent and often override simple isolation by distance (Sexton et al. 2014). This suggests an important role of ecological divergence during contemporary range expansions. However, the interplay of different environmental factors, phenotypic plasticity, gene flow, selection, and adaptation remains poorly understood, especially in the initial stages of contemporary range expansions.

Spiders provide some ideal model species to study the contribution of these factors to successful range expansions. Many spiders are efficient long‐distance dispersers. By passive wind‐mediated dispersal on silk threads, so called ballooning, they are capable of moving large distances in a single generation (Foelix 2010). They can thus quickly follow changing environmental conditions as evidenced by several ongoing poleward range expansions (Hänggi and Bolzern 2006; Muster et al. 2008; Kumschick et al. 2011). Recent work also shows that such range expansion can be accompanied and facilitated by rapid adaptive divergence of expanding lineages (Krehenwinkel and Tautz 2013). Spiders are also subject to intense parasitism by hymenopterans and dipterans. By causing more than 50% mortality in some populations, these parasites may impose strong selection (Finch 2005). A reduction of parasite load in novel environments could then result in rapid population growth.

The yellow sac spider, *Cheiracanthium punctorium* (Villers, 1789), offers an interesting example of an expanding spider species. It is a comparably large, free ranging, and nocturnal hunter, living on fallows and meadows. Their historical European distribution was largely limited to warm climate regions in southern Europe with the southern Upper Rhine Plain marking the northern limit of its distributional range (Bellmann 2006). Beginning in the mid‐20th century, isolated occurrences have been reported from more northern latitudes in the eastern German plains and as far north as Sweden (Muster et al. 2008). These novel occurrences were geographically restricted to small, isolated sites. However, over the last decade, a marked expansion has been noted from these focal areas and the species is now found in very high abundances in most of northeastern Germany, southern Sweden, and the Baltic States. At the same time, large gaps are present between these sites, in which the spider is currently not found, for example, Poland, northwestern Germany, and Denmark (Muster et al. 2008; Jonsson 2005; Krehenwinkel, pers. observation, Talvi and Rutkowski pers. communication). The spread of *C. punctorium* is of special relevance, as it is one of very few Central European spider species whose venom can cause noteworthy effects on humans and may require medical treatment (Muster et al. 2008).

Here, we aim to identify factors associated with the expansion success of the species in Europe. In particular, we hypothesize that genetic structure might reflect a pattern of environmental isolation between native and expanding populations, possibly as a result of rapid adaptation. Moreover, we hypothesize that a release from brood parasitism might be associated with range expansion success. Finally, we predict the species' potential distribution using species distribution modeling, highlighting its future expansion in Europe.

To test these hypotheses, we analyzed 400 *C. punctorium* specimens from several native and two invaded locations throughout the species' European range. We used a set of microsatellite loci, nuclear 28S rDNA, and mitochondrial COI sequences, to infer genetic structure and signatures of isolation. We also employed morphological measurements to show phenotypic differentiation of lineages. In addition, we estimated the frequency of eggsac parasitism in selected native and historical expanding populations to gather insights in the role of parasite release during the range expansion. Finally, we use multivariate comparisons between the realized niches of native and expanding populations to predict the species' historical and current potential distribution and to identify climatic factors associated with genetic differentiation of lineages and expansion success.

## Material and methods

### Sampling and morphology

We include 399 samples spanning most of the European range of *C. punctorium*. Our sampling of the native range covers southwestern Russia, eastern Ukraine, Slovenia, southwestern Germany, the Iberian Peninsula, and Italy. These sites comprise the historical native range of *C. punctorium*. In addition, we analyze specimens from two expanding foci: one in the Baltic States Latvia and Estonia and the other in the northeastern German state Brandenburg. Expanding populations were identified based on recent reports of novel occurrences or massive expansion from previously known, isolated occurrences (Muster et al. 2008; Jonsson 2005; Krehenwinkel pers. obs.; Tallvi, pers. comm.). The Baltic populations have only been recently identified and have probably been established less than a decade ago. Brandenburg constitutes an older population, which was first recorded in the mid‐20th century (Muster et al. 2008; Jonsson 2005; Krehenwinkel pers. obs.). Specimens were sampled by hand or sweep net. Additional samples were acquired from the Senckenberg Museum of Natural History. All mature female specimens were examined under a Leica dissecting microscope, and two morphological measurements were taken using a Leica measuring eyepiece (Leica, Wetzlar, Germany): prosoma width (at its widest part) as a measure of body size, and the length of the first leg's longest segment, the femur. As leg length relates to body size, we used relative leg length as ratio of femur length and prosoma width. We took the morphological measurements for 381 specimens from the Iberian Peninsula, Italy, Slovenia, Russia, northeastern Germany, and the Baltic states. While it is easy to quantify, body size is a trait of major ecological importance (Gardner et al. 2011). Moreover, body size has been shown to rapidly evolve in range expanding arthropods (Huey et al. 2000; Krehenwinkel and Tautz 2013).

### n‐dimensional hypervolumes and potential distribution

We compiled all available occurrence data for the species in Europe from the GBIF database (Dooley 2002), classical and online publications, a survey among European arachnologists, and by own field studies. In total, our distribution dataset comprises 228 records. Due to its notoriety as a venomous species, the occurrence and expansion of *C. punctorium* is quite well documented and new occurrences are usually accompanied by media coverage (Muster et al. 2008). Our approach thus probably allowed a reasonably accurate approximation of the species' contemporary range. To identify ecological differences between old and recently established populations, we distinguished native and expanding populations. Seventy‐three occurrences could be identified as native, 136 as expanding, while 19 occurrences had uncertain status. To distinguish native and expanding populations, we performed two separate analyses. A first analysis was performed with a reduced dataset of populations, for which we could acquire genotyping results (see Molecular analysis). Population subdivision was based on genetic differentiation. Based on these results, we performed a second analysis, assigning populations to the native and expanding groups based on proximity to genotyped populations and considering the species' documented expansion. Expanding sites were assigned to those, where a recent expansion of the species has been reported, or where it was only recently discovered. The results of both analyses were compared, to ensure a proper population assignment.

Based on GPS coordinates, we estimated the potential distribution of the species in Europe using the *hypervolume* package in R (R Development Core Team 2008; Blonder et al. 2014; Blonder 2015). We used 19 gridded bioclimatic variables with a spatial resolution of 2.5 arc min (approximately 4 km within Europe) representing average climate conditions between 1950 and 2000 from the WorldClim database (Hijmans et al. 2005). These 19 variables were transformed into four principal components with eigenvalues >1, which were used as orthogonal niche axes to compute the hypervolumes (for factor loadings, see Fig. S2).

The hypervolume analysis estimates the realized niche space of a species based on a random sample derived from multivariate kernel density estimates of the species' occurrence records in (Blonder et al. 2014). Two different approaches for the delimitation of the respective hypervolume are available: the first approach uses a bandwidth (herein = 1) enclosing the species records in environmental space (termed BWD hereafter), whereas the second approach delimits the realized niche space based on a multivariate minimum convex polygon (termed MMCP hereafter, see Supplementary Material S4 for a visualization of both approaches). Based on the hypervolumes of two taxa (herein native and expanding populations, as well as a set of records which could not be assigned to either of them), it is possible to compute various statistics including the volume of the realized niches per taxon, the intersection of two taxa as well as the unique volumes, the centroid distance between two taxa, and the Sorensen index [i.e., for hypervolumes A and B: S = 2*|A int B| / (|A| + |B|), Blonder 2015]. Furthermore, an inclusion test allows a projection of the hypervolumes into geographic space, representing the potential distribution of the species based on the estimates of the realized niches.

### Molecular analysis

One leg of each specimen was removed with sterile forceps, and genomic DNA extracted using the Qiagen DNeasy kit, according to the manufacturer's protocol (Qiagen, Hilden, Germany). A total of nine Iberian and nine Baltic DNA samples were chosen each from three populations, and their concentration measured on a NanoDrop Fluorospectrometer (NanoDrop, Wilmington, DE, USA). Equal DNA amounts were pooled for the two geographic regions, and each pool sequenced in 300 bp paired reads on one flow cell of an Illumina MiSeq. Library preparation and sequencing were conducted according to the manufacturer's protocols (Illumina, San Diego, CA, USA). The resulting DNA reads were quality trimmed with a minimum quality of 20 using Popoolation (Kofler et al. 2011). A de novo assembly was then generated including both libraries and using CLC genomic workbench with a word size of 45, a bubble size of 98, a minimum contig length of 1000 and including a scaffolding step (CLC Bio, Boston, MA, USA). We identified microsatellite markers in the genome assembly using Tandem Repeats Finder (Benson 1999). We then established a set of 14 polymorphic markers, which were genotyped for 260 specimens from all studied populations. Marker isolation, primer design, PCRs and genotyping were performed as described in Krehenwinkel and Tautz (2013). Microsatellite alleles were called using GeneMapper (Applied Biosystems, Foster City, CA, USA). Microsatellite analyzer (MSA) was used to generate population distance matrices (Nei's genetic distance) with 500 bootstrap replicates for the microsatellite data and to estimate genetic diversity (Dieringer and Schlötterer 2003). A neighbor‐joining tree was calculated based on the distance data with Phylip (Felsenstein 2002) and edited in MEGA 6. Pairwise *F*_ST_ values between populations were calculated in Genepop on the web (Raymond and Rousset 2002). In addition to the distance based analysis, we performed genetic clustering analysis using STRUCTURE (Pritchard et al. 2000; Falush et al. 2003). STRUCTURE was run with an admixture model, k‐values from 1–10, 10 replicates per k‐value, a burnin period length of 100 000 and 100 000 MCMC replications after burnin. The number of populations was estimated with STRUCTURE HARVESTER (Earl and von Holdt 2011) using the approach described in Evanno et al. (2005). We performed two independent STRUCTURE analyses: one with all sampled populations and one with only native European populations, to exclude potential masking effects by highly divergent expanding populations.

Approximately 900 bp fragments of the 28S rDNA (for 105 specimens) and the mitochondrial COI gene (for 399 specimens) were PCR amplified using the Qiagen Multiplex PCR kit according to the manufacturer's protocol and using the primers 28Srd4.8a and 28Srd7b1 (Schwendinger and Giribet 2005) and C1‐J‐1718‐spider and C1‐N‐2776‐spider (Vink et al. 2005). While COI is well known as a useful phylogeographic and taxonomic marker in spiders (Krehenwinkel and Tautz 2013), the more slowly evolving 28S might also be suited to uncover interspecific divergence in spiders (Jäger et al. 2015). We thus use it as a backup for the COI data here. Sequence traces were edited using Codon Code Aligner (Codon Code Corporation, Centerville, MA, USA) and aligned in MEGA 6 (Tamura et al. 2013) under default alignment parameters. The mitochondrial sequences were translated to amino acids to detect potential presence of pseudogenes.

Next, we aimed to identify those climatic factors, which directly relate to genetic divergence and thus possibly contribute to divergence or cohesion of lineages across Europe. The association of the 19 bioclimatic variables to genetic differentiation was evaluated using GESTE (Foll and Gaggiotti 2006). In addition to the bioclimatic variables, we included longitude and latitude for all populations in the analysis, to test for isolation by distance. GESTE was run with 10 pilot runs and a sample size of 10 000, with a thinning interval of 20 and a burnin of 50 000.

### Parasite infection

After mating, *C. punctorium* females build silken retreats on top of grass stalks, in which they construct a single eggsac and guard the hatching offspring till they die in late autumn. The guarding female will attack intruders ferociously (Bellmann 2006). Nevertheless, we found numerous eggsacs to be infected by larvae of a parasitic dipteran that consume all spider eggs before hatching. Infection with these parasites thus leads to a complete reproductive failure of the female. We screened nine native populations in Italy, and ten expanding populations in the Baltic States and northeastern Germany for the presence and abundance of this parasite. Moreover, we gathered information from arachnologists working in the respective areas. To screen for parasites, 10–20 female retreats per site were opened and eggsacs inspected for parasitic larvae. Some larvae were taken to the laboratory where COI barcodes were generated for identification of the species using standard arthropod barcoding primers (Folmer et al. 1994). PCR and sequencing were conducted as described above. The resulting sequences were blasted against the GenBank database, to identify related sequences.

## Results

### Nuclear microsatellite data

**Figure 1:**
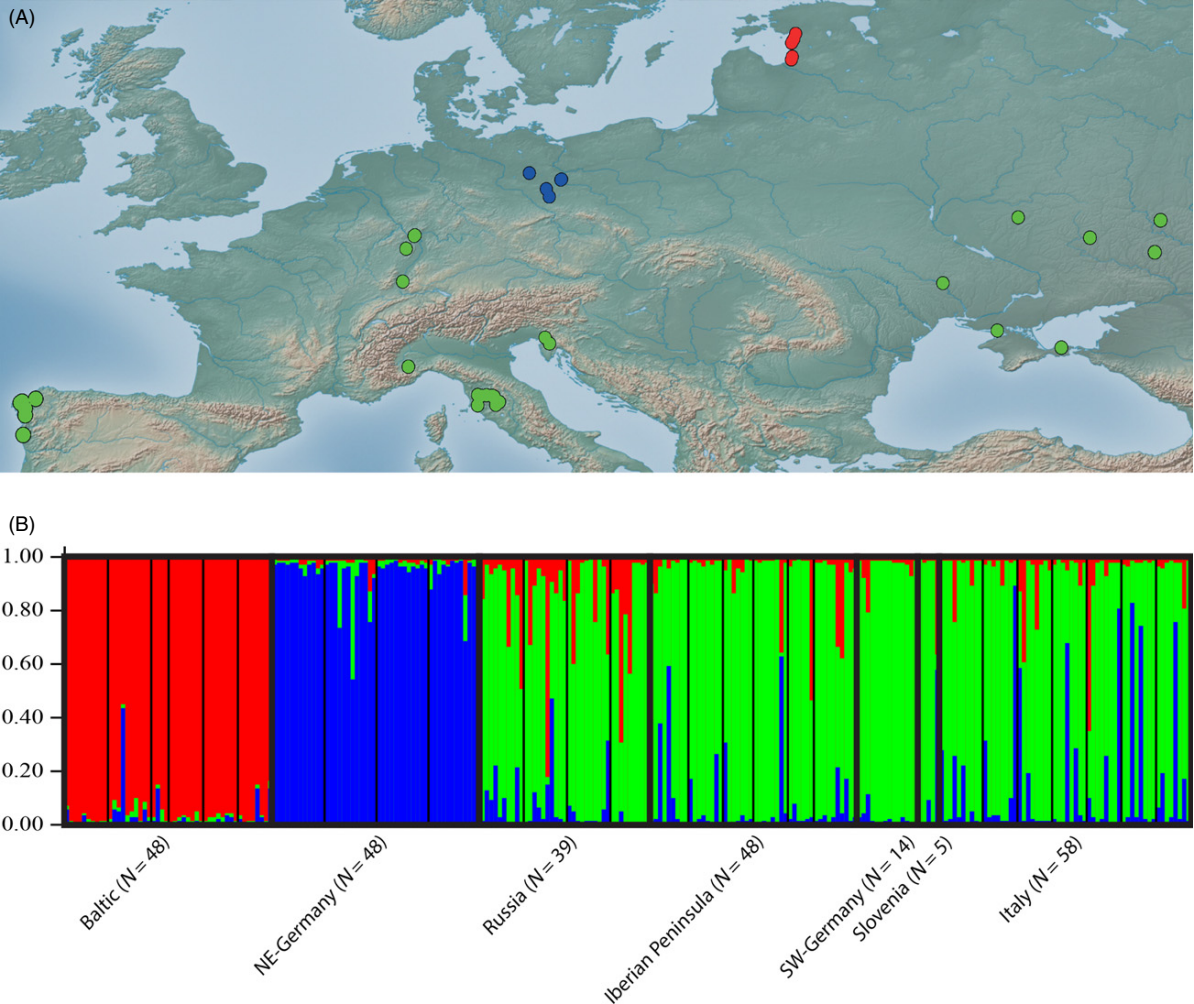
(A) Sampling map for the analyzed yellow sac spider populations, showing native populations in green, expanding northeastern German ones in blue and expanding Baltic ones in red. B. Result of a STRUCTURE analysis assuming K = 3 and based on 14 microsatellite loci for the according populations. Colors correspond to those in Fig. 1A.

The MiSeq run yielded ~14*10^6^ sequences for the Iberian DNA pool and ~20*10^6^ for the Baltic one. The genome assembly amounted to 148 601 contigs in ~ 236*10^6^ bases, with an N50 of 1425 of which we could isolate primers for 4453 microsatellite loci. We genotyped populations across the European range of the yellow sac spider (Fig. 1A) for 14 of these loci. This revealed significant genetic structure. Our STRUCTURE analysis suggested k = 3 populations. We found a homogeneous native European population, including samples from the Iberian Peninsula, Italy, Slovenia, southwestern Germany and Russia (Fig. 1B). In contrast to the homogeneity in the native range, we identified two genetically separated expanding populations: one in the Baltic States and another in the northeastern German state of Brandenburg. The northeastern German populations were less divergent in comparison with the native cluster than the Baltic ones (allele frequency difference Baltic versus. Native = 0.045, allele frequency difference NE‐Germany versus Native = 0.022). Moreover, the two expanding clusters were clearly divergent (allele frequency difference Baltic versus NE‐Germany = 0.034). A second STRUCTURE analysis excluding the two expanding populations suggests additional and finer‐scaled genetic structure in the native European cluster with k = 2. Eastern and western European populations from Russia and the Iberian Peninsula each form separate clusters. The eastern cluster extends to the Balkans and southwestern German, while populations from Italy appear admixed between these two clusters (Figure S2). These results were generally supported by an allele sharing phylogeny of the data with the expanding population from northeastern Germany and the Baltic States forming well‐separated clades. The phylogeny also suggested a similar differentiation of Russian and Iberian populations within the native range, with other native populations grouping between these two (Figure S3). An analysis of *F*_ST_ differentiation between populations additionally confirmed our previous analyses. The two expanding populations were divergent from each other and all native populations, while the differentiation between native populations was considerably lower (Table 1).

**Table 1:** Pairwise *F*_st_ divergence of the five main populations in our study

	Iberian Pi	NE‐Germany	Baltic	Italy
NE‐Germany	0.061	–	–	–
Baltic	0.067	0.076	–	–
Italy	0.030	0.038	0.054	–
Russia	0.039	0.048	0.047	0.013

We did not find a pronounced difference of genetic diversity between native and expanding spider populations. This held true for allelic richness (AR) and expected heterozygosity (EH). The highest diversity was found in Russia (AR = 3.69, EH = 0.63), followed by Iberia (AR = 3.46, EH = 0.60) and Italy, then northeastern Germany (AR = 3.31 3.62, EH = 0.58), and finally, the lowest in the Baltic States (AR = 2.85, EH = 0.51). The only significant difference in diversity was found for the Baltic populations, which showed a reduction of allelic richness and expected heterozygosity compared to all other populations (anova, Games–Howell post hoc test, *P *<* *0.01). Russian, Iberian, and populations from northeastern Germany did not show significant differences in diversity.

To summarize, our genetic analyses identified a fairly homogenous native European population from Russia over Slovenia and Italy to southern Germany and the Iberian Peninsula. Only slight differentiation between the eastern and western native range is evident. This was contrasted by two divergent expanding populations in northeastern Germany and the Baltic states. We did not find a consistent signature of reduced genetic variation in recently established populations compared to native ones.

### Mitochondrial COI and nuclear 28S rDNA sequences

**Figure 2:**
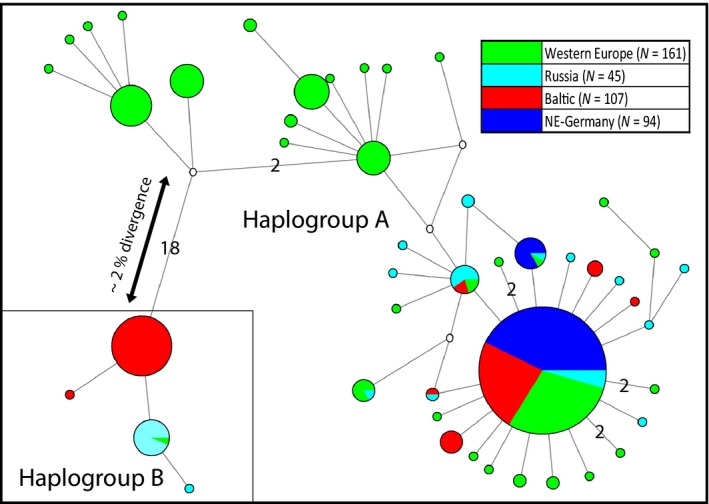
Median‐joining mitochondrial haplotype network for ~850 bp of the COI gene in 399 specimens from native and expanding European yellow sac spider populations. Unless indicated by numbers, branches between haplotypes imply single substitutions. White circles represent hypothetical, unobserved interconnections between haplotypes.

The mitochondrial haplotype network (Fig. 2) showed a dumbbell pattern of two divergent haplogroups, separated by 18 substitutions (groups A and B). Haplogroup A showed several closely related starburst patterns of haplotypic radiations, with most haplotypes divergent by 1–3 substitutions. In contrast, haplogroup B was quite homogeneous and consisted of only four haplotypes. Haplogroup A was found throughout the spider's range. Native European populations from the Iberian Peninsula, Italy, Slovenia and southwestern Germany almost all exclusively carried haplotypes of group A. The distribution of haplotypes within these native populations did not show pronounced geographic structure. A single Iberian specimen carried a haplotype of group B. This same haplotype was found at a much higher frequency in Russian populations (about 30%). Considering the high divergence between haplogroups A and B, Russian populations showed a higher nucleotide diversity than other native ones (*π*_Russia_ = 0.0122_,_ *π*_Iberia_ = 0.0036, *π*_Italy_ = 0.0038), while haplotype diversity was more comparable (HD_Russia_ = 0.841, HD_Iberia_ = 0.679, HD_Italy_ = 0.818). Similar to the Russian populations, about 30% of the specimens from the Baltic States also carried haplogroup B. Their haplotype was distinct from the Russian one by a single substitution. Their nucleotide diversity was high, while their haplotype diversity was reduced compared to native populations (*π*_Baltic_ = 0.0115_,_ HD_Baltic_= 0.617). An even more pronounced reduction of diversity was found for expanding populations in northeastern Germany, which carried only two slightly divergent haplotypes from group A (*π*_NE‐Germany_ = 0.0002_,_ HD_NE‐Germany_ = 0.189). In contrast to the mitochondrial data, the 28S rDNA was completely monomorphic across all of Europe, with all 105 sequenced specimens sharing the same 28S alleles.

### Morphology

**Figure 3:**
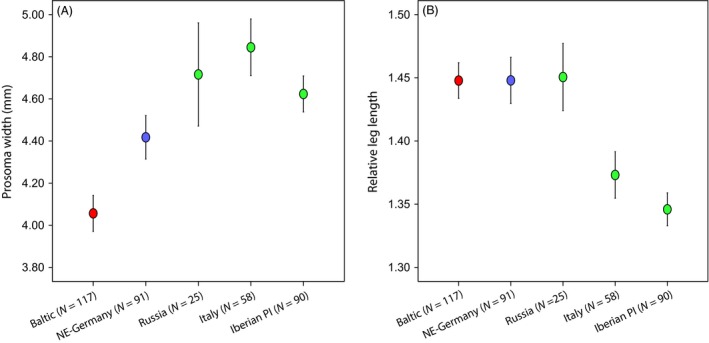
(A) Average body size (prosoma width) of mature females in native and expanding European yellow sac spider populations. (B) Relative leg length (femur I length / prosoma width) of mature females in native and expanding European yellow sac spider populations. Bars represent the 95% confidence interval of the mean; colors correspond to those in the sampling map in Fig. 1A.

Our body size measurements indicated a homogenous native population from Russia (average body size ± standard deviation = 4.72 ± 0.59 mm), Italy (4.82 ± 0.51 mm) and the Iberian Peninsula (4.62 ± 0.41 mm), as well as two isolated expanding ones in the Baltic states (4.06 ± 0.47 mm) and northeastern Germany (4.42 ± 0.50 mm) (Fig. 3A). Body size, as measured by the prosoma width, of native populations was not significantly different, while the expanding populations were each distinguished by a significantly smaller body size from the native populations (anova, Games–Howell post hoc test, *P* < 0.05). In addition, we found a significant size difference between the two expanding populations, with specimens from northeastern Germany being larger than those from the Baltic. Overall, the body size data were highly congruent with our microsatellite analysis.

The relative leg length showed slightly different results (Fig. 3B). Here, we could distinguish a group comprising western specimens from Iberia (average relative leg length ± standard deviation = 1.35 ± 0.06) and Italy (1.37 ± 0.07), which were distinct from other populations by significantly shorter legs (anova, Games–Howell post hoc test, *P *< 0.05). Russian spiders and those from the two recently colonized locations shared a very similar relative leg length (average relative leg length Russia = 1.45 ± 0.06, Baltic = 1.45 ± 0.08, northeastern Germany = 1.45 ± 0.09). This analysis thus corroborated a closer relationship of Russian and expanding populations.

### Parasite infections

DNA barcoding identified the eggsac parasite as *Sarcophaga sexpunctata* (Fabricius, 1805), a widely distributed flesh fly known to parasitize eggsacs of different spider species. We screened 10 expanding and nine native populations for *S. sexpunctata* infections. Of nine screened native Italian populations, all showed infections. We estimated the abundance of *S. sexpunctata* infections in four of these populations. Between 30% and 90% of all eggsacs were parasitized, on average 50%. At the same time, none of the 10 analyzed expanding populations from the Baltic States and northeastern Germany were affected by brood parasitism. Inquiries with fellow arachnologists did not reveal any incidence of parasitism in additional expanding populations in Sweden and northeastern Germany (Jonson and Friman pers. comm.).

### n‐dimensional hypervolumes, potential distributions, and GESTE analysis

Principal component analysis revealed four PCs with eigenvalues >1. PC1 was predominantly composed of temperature and precipitation‐related variables (Bio1: Annual Mean Temperature, Bio6: Minimum Temperature of the Coldest Month, Bio9: Mean Temperature of the Driest Quarter, Bio11: Mean Temperature of the Coldest Quarter, Bio13: Precipitation of Wettest Month, and Bio16: Precipitation of Wettest Quarter). PC2 was most strongly correlated with Bio14: Precipitation of the Driest Month and Bio18: Precipitation of the Warmest Quarter, while PC3 was driven by Bio2: Mean Diurnal Temperature Range, Bio4: Temperature Seasonality, and Bio7: Temperature Annual Range. PC4 represented mainly ‘Precipitation Seasonality’ (Bio15). For all factor loadings, see Supplementary Material S4.

**Figure 4:**
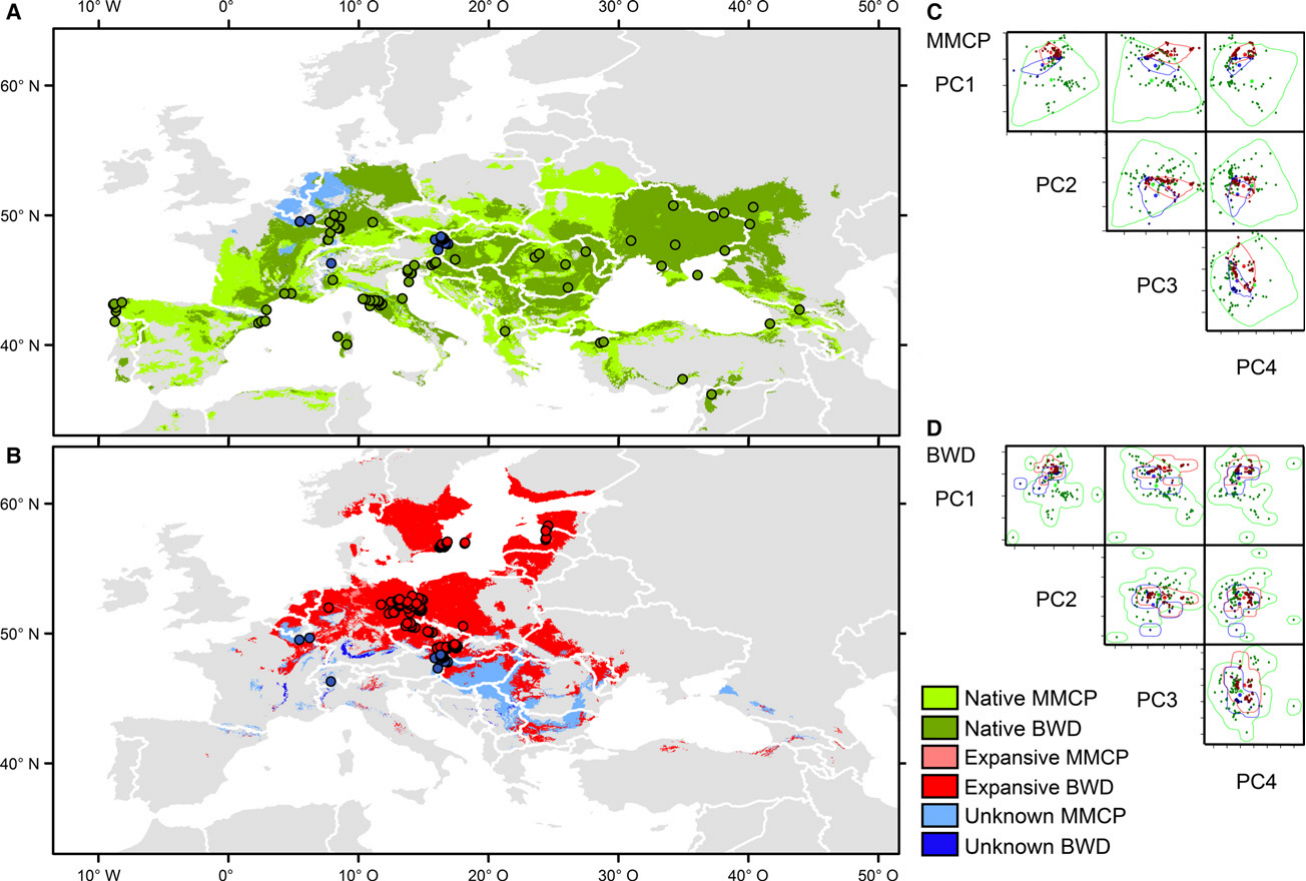
(A) Potential current distribution of yellow sac spiders in Europe, based on only native European populations as well as populations with unknown status. (B). Potential current distribution of yellow sac spiders in Europe, based on all currently known occurrences of expanding populations, as well as populations with unknown status. (C, D) Estimated hypervolumes based on a bandwidth approach (BWD) and multivariate minimum convex polygons (MMCP). For high‐resolution versions of the biplots, see Supplementary Material S4.

Based on a comprehensive set of species records, the realized climatic niche space of the native populations was much larger than the niche space occupied by the expanding populations and those whose status could not be determined (Volume _BWD Native_ = 3172.6; Volume _BWD Expanding_ = 19.7; Volume _BWD Unknown_ = 5.3; Volume _MMCP Native_ = 722.3; Volume _MMCP Expanding_ = 236.2; Volume _MMCP Unknown_ = 108.2). For visualization, see Fig. 4 and Supplementary Material S4. The shared volumes between native and expanding populations were comparatively small (Intersection _BWD Native_ _/ Expanding_ = 57.0; Intersection _MMCP Native / Expanding_ = 6.4) leading to low–very low Soerensen indices (S _BWD Native_ _/ Expanding_ = 0.12; S _MMCP Native / Expanding_ = 0.004). This indicates a strong niche shift between native and expanding populations, wherein the combination of PC1 and PC3 had the highest contribution to the differentiation (Fig. 4).

The potential distributions derived from the hypervolumes indicated a much wider distribution for the species than currently known. This holds especially true for northern European expanding populations. While the species is currently only known from isolated foci, our model indicates a potential distribution over Poland, northern Germany, and the countries around the southern Baltic Sea (Fig. 4).

An association of genetic structure with bioclimatic variables and longitude and latitude was tested using GESTE. The highest probability model of our GESTE analysis suggests an association of only Bio10 (Mean Temperature of Warmest Quarter) with genetic structure over Europe (posterior probability = 0.228, marginal probability = 0.568, *α *= −0.63). For the analyzed populations, the genetic differentiation decreases with increasing summer temperatures (Fig. S1). In contrast, neither geographic distance nor winter cold explain the observed genetic differentiation.

## Discussion

### Global change, range expansions, and environmental isolation of expanding populations

Contemporary range expansions are often simply attributed to global change (Hickling et al. 2006), but instead might be caused by a complex interplay of factors. A combination of global change, ecological processes, phenotypic plasticity, and finally adaptation might pave the way for the currently observed massive expansions of many species (Gienapp et al. 2008; Franks and Hoffmann 2012). This might also hold true for the yellow sac spider. Warming climate and the increasing availability of fallow land could have initially enabled an expansion into novel habitats. The expansion did not occur on a broad front. Instead, several suitable patches of habitat were colonized outside of the native range. First isolated occurrences of the yellow sac spider were in fact reported from northeastern Germany in the mid‐20th century (Muster et al. 2008). Like many other spiders, *C. punctorium* probably possesses high dispersal ability, due to passive, wind‐mediated ballooning of young spiderlings (Bell et al. 2005; Krehenwinkel et al. 2016). The range wide homogeneity of 28S rDNA and overall low microsatellite differentiation within the native range also suggests connectivity of populations by gene flow. Suitable emerging habitat and changing temperatures can probably be quickly reached by the species.

Our genetic analyses suggest two independent colonization events: one into northeastern Germany and one into the Baltic States. Our genetic and morphological analyses suggest similarity between Russian and both expanding populations. An important source of expanding populations might be located in eastern European steppe. The climate in this region is distinguished by cold winters and thus more comparable to that in the newly invaded *C. punctorium* habitats. The invading populations could therefore be preadapted to the novel conditions, which might facilitate their initial establishment.

The observation of two divergent mitochondrial haplotypes in the Russian and Baltic expanding populations resemble recent findings in *Argiope bruennichi*, another expanding spider species (Krehenwinkel and Tautz 2013; Krehenwinkel et al. 2015). Here, an admixture of two formerly separated lineages relates with rapid divergence of expanding and native populations. This finding is in line with many recent studies that associate expansion success and genetic admixture (Kolbe et al. 2004; Nolte and Tautz 2010; Rius and Darling 2014). Interestingly, our microsatellite analysis suggests the contact of a western and eastern European native genetic cluster in Central Europe. Expanding populations might thus have been founded from admixed populations. However, a deeper geographic sampling and more genetic data will be necessary to quantify an association of admixture and expansion.

While the source of expanding populations might be preadapted to cold winters, expanding spiders also have to cope with cooler and more humid summers in northern Europe. The environmental isolation of expanding populations could be explained by divergent selection between the newly colonized and native environments. The time lag from the initial establishment in the mid‐20th century to the current expansion might be explained by the necessary waiting time for adaptations to emerge (Clements and Ditommaso 2011). Such lag phases are commonly observed in biological invasions and range expansions (Sexton et al. 2009). An adaptation to novel climatic regimes has been shown to evolve quickly in spiders, even in the face of high gene flow (Tanaka 1996; Krehenwinkel and Tautz 2013). Apart from direct climate tolerance, a particular phenotype in northern European populations could be found in their reduced body size. Yellow sac spiders are an annual species. They grow from spring till summer and then reproduce. A reduced body size might thus be an adaptation to a shorter growing season in cooler climates and a necessity for earlier reproduction in more northern latitudes. Latitudinal size gradients have been described for many arthropod species and can evolve quickly (Huey et al. 2000; Krehenwinkel and Tautz 2013). However, more experimental data, for example, from reciprocal transplant experiments, common garden studies and whole genome sequencing, will be necessary to support the assumption of an adaptive divergence of the studied populations. Based on our data, we currently cannot exclude an involvement of phenotypic plasticity in the observed differences. Most phenotypes are known to be affected by environmental context, and plasticity plays an important role in species responses to contemporary climate change (Gienapp et al. 2008; Merilä and Hendry 2014).

### A parasite release in expanding populations

The species' range expansion might be facilitated by a release from brood parasitism in the recently colonized range (Menéndez et al. 2008; Phillips et al. 2010). By effectively removing 50% of each generation in native populations, *Sarcophaga sexpunctata* imposes a very strong mortality factor. Interestingly, *S. sexpunctata* is widely distributed in Europe and feeds on eggs of different spider species (Povolný 2000), yet, according to our results, does not parasitize on the expanding populations of *C. punctorium*. Northern European *S. sexpunctata* populations might require more time to adopt *C. punctorium* as a novel food source. Moreover, *S. sexpunctata* has been considered thermophilic and might show a decreasing population density with increasing latitude (Povolný et al. 2003).

### Massive expansion of a venomous spider over Europe

Yellow sac spiders are currently massively spreading over northern Europe. Our species distribution model suggests a much wider potential distribution of *C. punctorium* in northern Europe than currently observed. We predict a colonization of most of Germany, the Baltic States, Poland, Denmark, and southern Sweden. The yellow sac spider is the only venomous Central European spider whose bites frequently necessitate medical treatment. A yellow sac spider bite can be very painful and might even cause side effects such as nausea, vertigo, fever, and shivers (Sacher 1990; Weimann et al. 2011; Papini 2012; Nentwig et al. 2013). Although the *C. punctorium* venom is not life threatening and its bites are rare even in densely colonized areas (Sacher 1990), our predictions are nevertheless important, in that they highlight one of the consequences of global change. In addition to increasing reports of disease vectors expanding their ranges (Khasnis and Nettleman 2005), another problematic outcome of global change is the spread of venomous species into new territories.

## Conclusions

Our study provides evidence for rapid genetic and ecological differentiation of expanding populations during a contemporary range expansion. This differentiation might be caused by adaptive divergence, which could additionally fuel the success of the expansion. However, more experimental data (e.g., from reciprocal transplants or genome sequencing) will be required to rule out phenotypic plasticity as the driving force of the observed expansion. In the coming decades, an unprecedented expansion of the venomous yellow sac spider into northern Europe is expected, highlighting one of the health‐related perils of the ongoing climatic changes.

## Data archiving statement

The following data are available online from: GenBank:

1. COI sequences and 28S rDNA sequences Dryad (http://dx.doi.org/10.5061/dryad.2q8p6):
2. Georeferenced sampling sites and records used for the distribution model
3. COI and 28S rDNA sequence alignments
4. Microsatellite data and Microsatellite primers
5. Morphological measurements for all specimens
6. Genome assembly.

## Supporting information


**Figure S1**. Association of genetic differentiation and bioclimatic variable Bio10.
**Figure S2**. Result of a STRUCTURE analysis of only the native populations and assuming k = 2 and based on 14 microsatellite loci.
**Figure S3**. Unrooted neighbor joining phylogeny of European yellow sac spider populations, based on Nei's genetnei'sic distance of 14 microsatellite loci.
**Figure S4**. A. Potential current distribution of yellow sac spiders in Europe, based on only genotyped native and expansive European populations. B. Estimated hypervolumes based on a bandwidth approach (BWD) computed only for genotyped native and expansive European populations. C. Estimated hypervolumes based on multivariate minimum convex polygons (MMCP) computed only for genotyped native and expansive European population.
