# Supplementary material for: Rapid genetic and ecological differentiation during the northern range expansion of the venomous yellow sac spider Cheiracanthium punctorium in Europe
Source: Evol Appl. 2016 Aug 17;9(10):1229–40. doi: 10.1111/eva.12392 (PMC5108215; doi:10.1111/eva.12392)
Supplement: Supplementary file 1 — Figure S1. Association of genetic differentiation and bioclimatic variable Bio10. Figure S2. Result of a STRUCTURE analysis of only the native populations and assuming k = 2 and based on 14 microsatellite loci. Figure S3. Unrooted neighbor joining phylogeny of European yellow sac spider populations, based on Nei's genetnei'sic distance of 14 microsatellite loci. Figure S4. A. Potential current distribution of yellow sac spiders in Europe, based on only genotyped native and expansive European populations. B. Estimated hypervolumes based on a bandwidth approach (BWD) computed only for genotyped native and expansive European populations. C. Estimated hypervolumes based on multivariate minimum convex polygons (MMCP) computed only for genotyped native and expansive European population. [file EVA-9-1229-s001.docx]

**Supporting information**

**Figure S1** Association of genetic differentiation and bioclimatic variable Bio10 (Mean Temperature of the Warmest Quarter), as found for the most probable model of our GESTE analysis of European yellow sac spider populations.


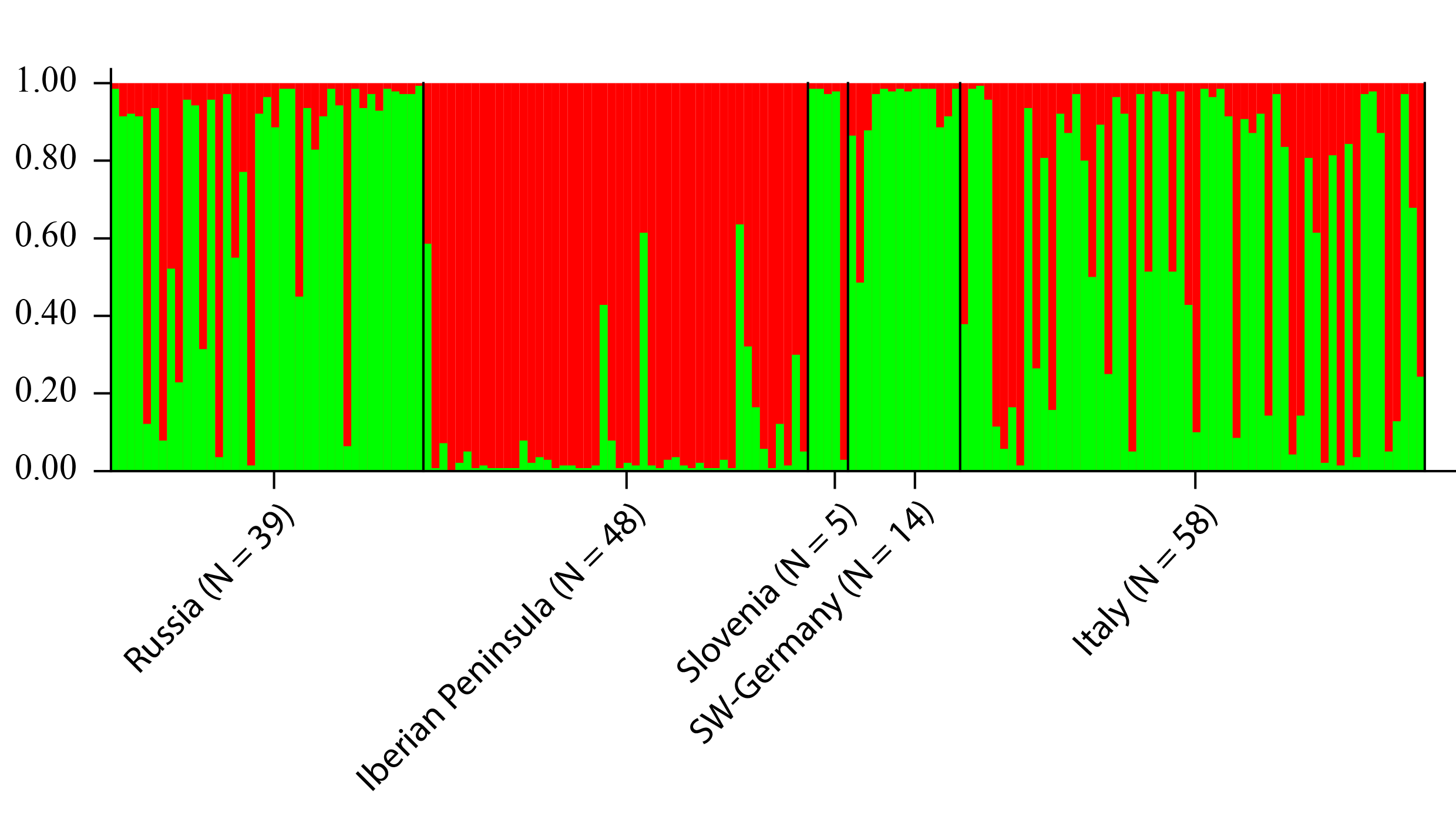


**Figure S2** Result of a STRUCTURE analysis of only the native populations and assuming k = 2 and based on 14 microsatellite loci.


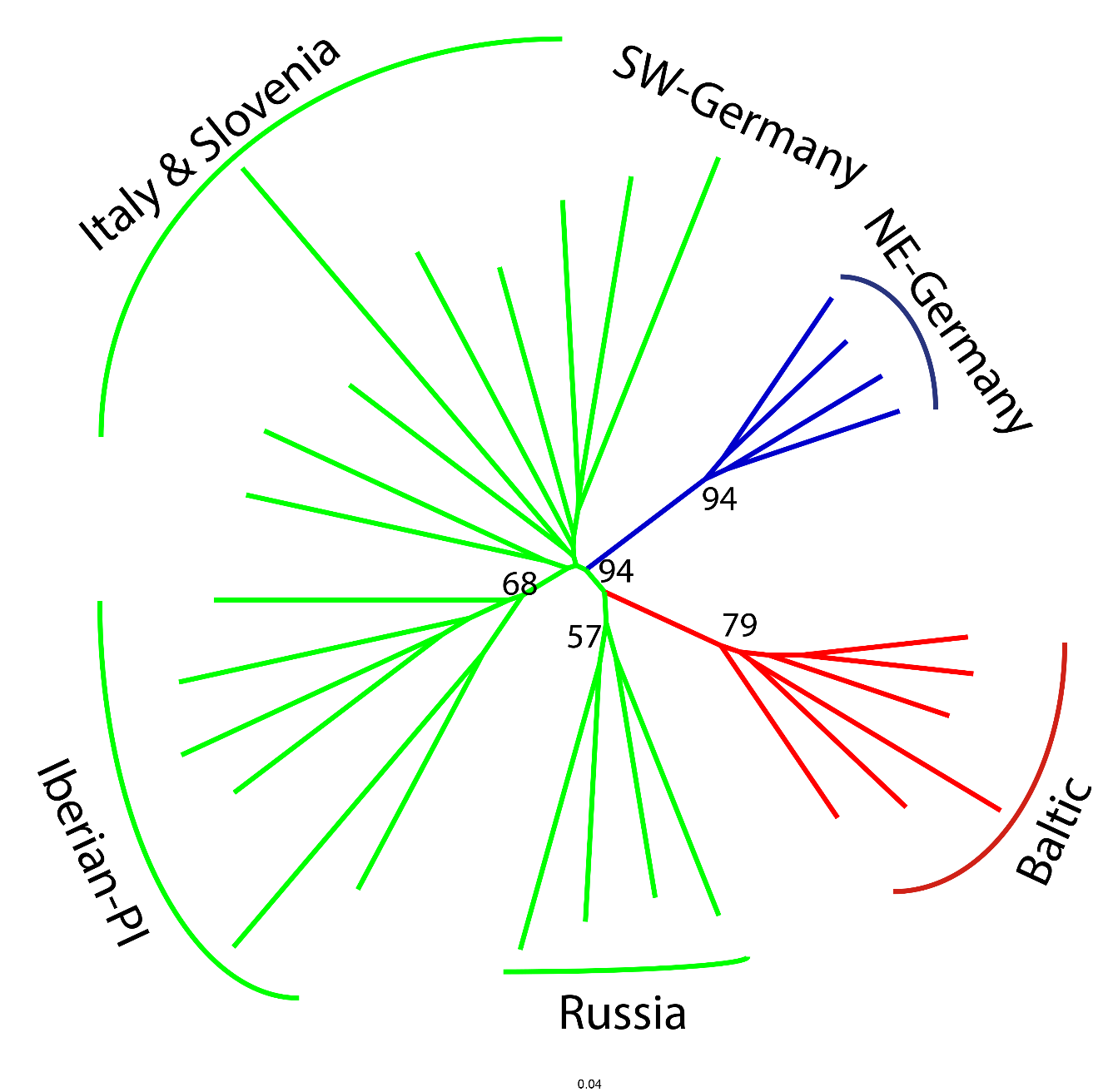


**Figure S3** Unrooted neighbor joining phylogeny of European yellow sac spider populations, based on Nei’s genetnei’sic distance of 14 microsatellite loci. Colors correspond to those in Fig. 1A. Bootstrap support for all major braches is shown after 500 bootstrap replicates.

**Supplementary Material S4**

Based on a pruned set of species records including only genotyped populations, the realized climatic niche space of the native populations was much larger than the niche space occupied by the expanding populations (Volume _BWD Native_ = 275.0; Volume _BWD Expansive_ = 61.9; Volume _MMCP Native_ = 232.1; Volume _MMCP Expansive_ = 0.1). For visualization see Fig. S4. The shared volumes between native and expanding populations were comparatively small (Intersection _BWD Native_ _/ Expansive_ = 13.7; Intersection _MMCP Native / Expansive_ = 0) leading to low - very low Soerensen indices (S _BWD Native_ _/ Expansive_ = 0.08; S _MMCP Native / Expansive_ = 0.00). This indicates a strong niche shift between native and expanding populations, wherein the combination of PC1 and PC3 had the highest contribution to the differentiation (Fig. S4).

As when trained with the full set of species records the potential distributions derived from the hypervolumes indicated a much wider distribution for the genotyped populations than currently known. However, some parts of the climate space of Central and Eastern Europe are missing.


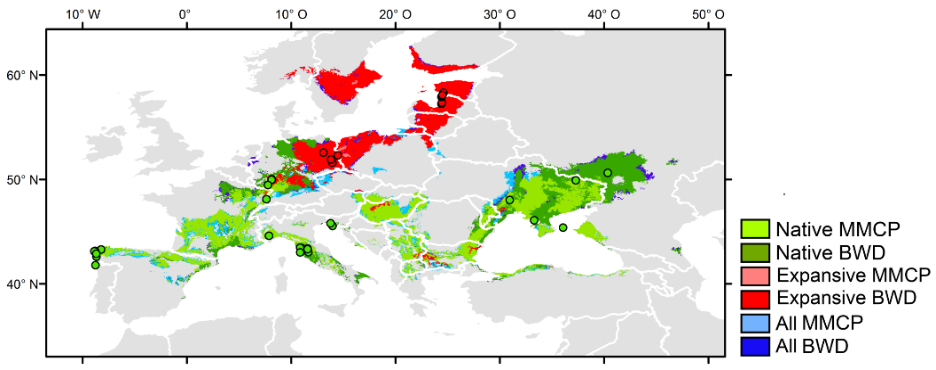


**Figure S4 A.** Potential current distribution of yellow sac spiders in Europe, based on only genotyped native and expansive European populations.


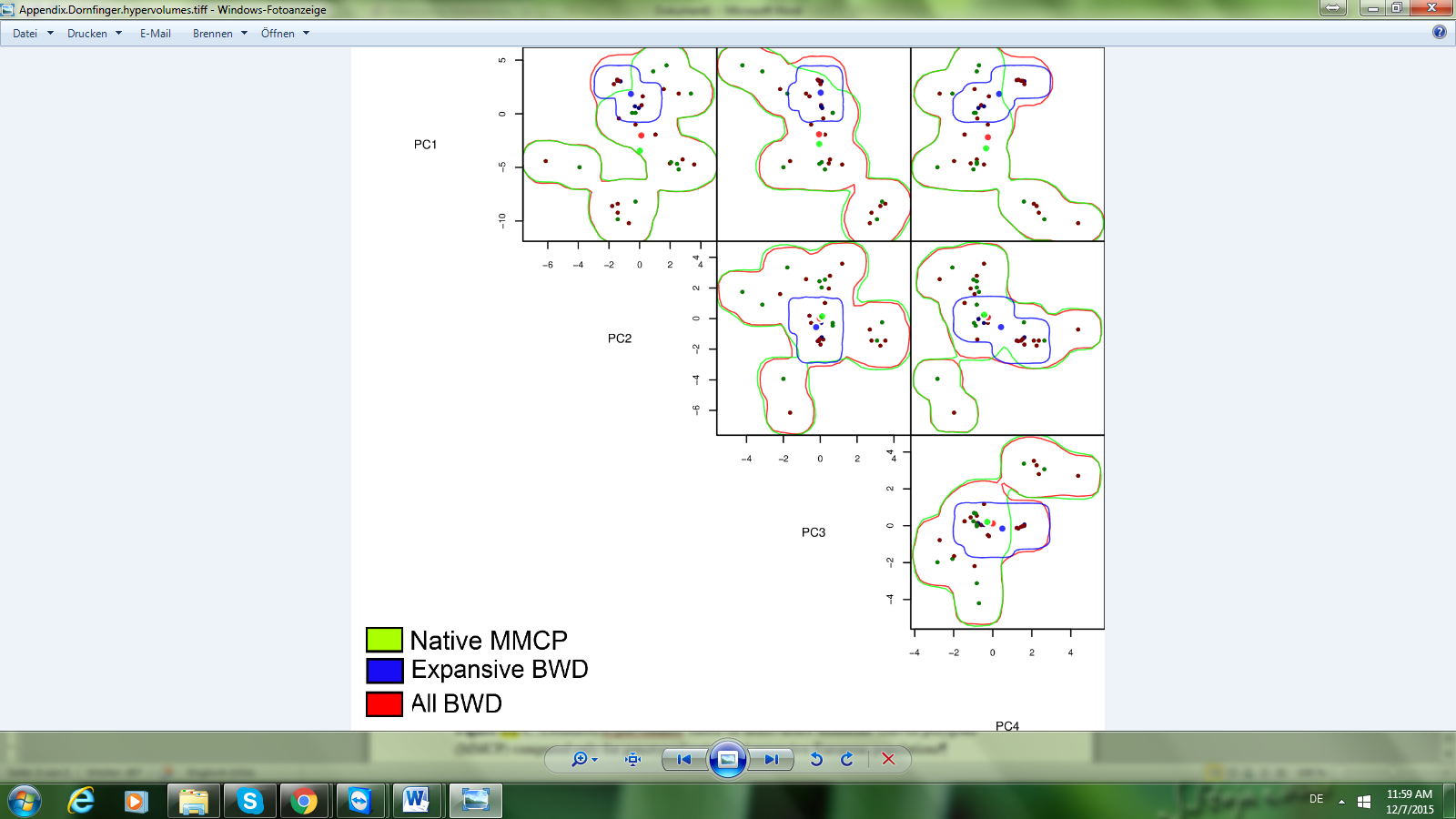


**Figure S4 B.** Estimated hypervolumes based on a bandwidth approach (BWD) computed only for genotyped native and expansive European populations.


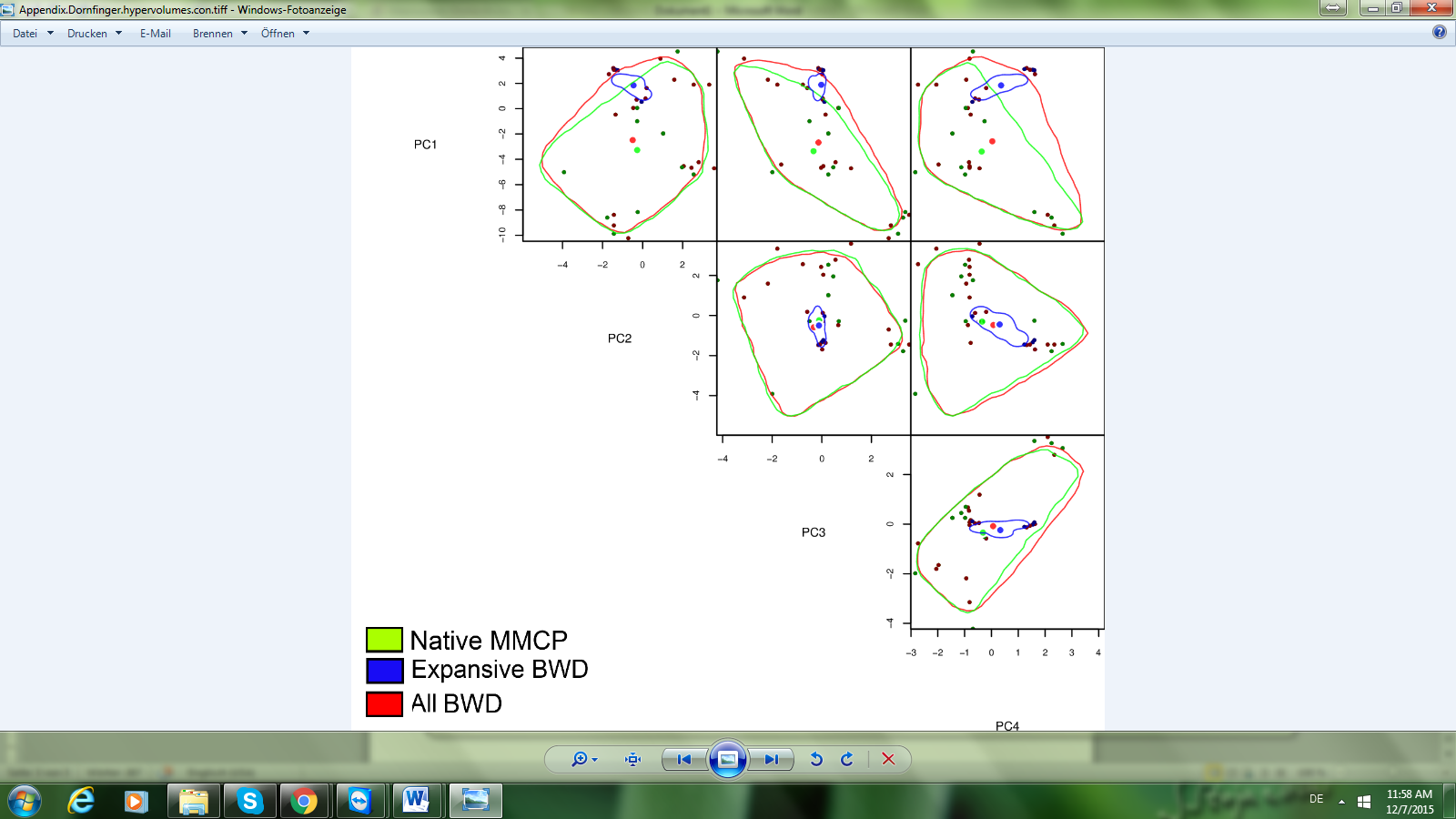


**Figure S4 C.** Estimated hypervolumes based on multivariate minimum convex polygons (MMCP) computed only for genotyped native and expansive European population.
